# Assessing Public Awareness and Social Acceptance of Scoliosis Screening and Treatment Options in Saudi Arabia: A Nationwide Cross-Sectional Study

**DOI:** 10.3390/healthcare13233110

**Published:** 2025-11-28

**Authors:** Abdulmonem Alsiddiky, Abdulaziz S. AlNahari, Abdulmalik A. Alomrani, Mayssar Bassam Alshobaki, Naif Alateeq, Abdullah Nasser Almawash, Maha Emad Aldaijy, Sarah Essa Alsuwaidan, Mohammed Khalid Alqifari, Mohammed N. Aldawsari, Sara Alhomaidhi

**Affiliations:** 1 Department of Orthopedics, College of Medicine, King Saud University, Riyadh 11451, Saudi Arabia; alsiddiky@ksu.edu.sa; 2 College of Medicine, King Saud University, Riyadh 11451, Saudi Arabia; abzsaad2017@gmail.com (A.S.A.); maloki3213@gmail.com (A.A.A.); mayssaralshobaki@gmail.com (M.B.A.); naif.alateeq443@gmail.com (N.A.); abdullahnassermed@gmail.com (A.N.A.); aldaijym@gmail.com (M.E.A.); sara.essa1423@gmail.com (S.E.A.); oamiq2001@gmail.com (M.K.A.); mn3@hotmail.fr (M.N.A.)

**Keywords:** scoliosis, awareness, school-based screening, treatment acceptability, public health, Saudi Arabia

## Abstract

**Background:** Scoliosis is a common musculoskeletal disorder that remains underrecognized in Saudi Arabia, where limited public awareness may delay early detection and treatment. This study assessed public knowledge, support for school-based screening, and attitudes toward treatment. **Methods:** A nationwide cross-sectional survey was conducted during the 2025 Saudi National Scoliosis Awareness Campaign through public venues and online platforms. A culturally adapted questionnaire assessed demographics, knowledge, symptom recognition, treatment preferences, barriers, and information sources. A total of 2055 responses were analyzed using descriptive statistics and chi-square tests. **Results:** While 70.9% of participants had heard of scoliosis, only 18.1% reported good understanding. Symptom recognition was limited, with spinal curvature, back pain, and uneven shoulders most often identified. Most respondents supported school-based screening (88.2%) and early detection (92.1%), with a mean preferred screening age of 8 years. Bracing (64.3%) and surgery (53.5%) were more accepted than observation (30.5%), though concerns about pain, effectiveness, and appearance were frequent. Social media was the most common initial information source (34.6%), while healthcare professionals were the preferred future source (79.2%). Reported barriers included lack of awareness (68.2%), difficulty accessing specialists (19.9%), and cost (8.9%). **Conclusions:** Public support for school-based screening is strong, but baseline knowledge remains limited, and treatment perceptions are shaped by psychosocial and cultural concerns. National campaigns, school curricula, and healthcare-led education, combined with accessible, low-cost screening, may enhance early detection and improve scoliosis management in Saudi Arabia.

## 1. Introduction

Scoliosis is a three-dimensional spinal deformity characterized by a lateral curvature of at least ten degrees, often accompanied by vertebral rotation and decreased thoracic kyphosis [1]. It can develop at any age, with idiopathic scoliosis being the most common form among children and adolescents, while degenerative scoliosis primarily affects older adults. Despite its clinical relevance, scoliosis remains an underrecognized musculoskeletal disorder both globally and in Saudi Arabia [2].

Previous studies in the Kingdom have demonstrated limited awareness regarding scoliosis etiology, early detection, and conservative treatment strategies [2,3]. This lack of public knowledge contributes to diagnostic delays and potentially avoidable disease progression. For instance, one national study revealed that nearly 98% of families were unaware of the optimal timing for congenital scoliosis surgery [2], while another identified scoliosis as among the most frequently overlooked conditions in school-based health screenings [3]. Such findings underscore persistent weaknesses in early detection and the absence of standardized national screening protocols.

Beyond clinical factors, cultural attitudes and social perceptions strongly influence health behavior and treatment decisions in Saudi Arabia [4,5]. Family dynamics, religious beliefs, and stigma surrounding visible deformities often determine whether individuals seek or adhere to medical care. In the context of scoliosis, these psychosocial factors can hinder both participation in screening programs and acceptance of treatments such as bracing or surgery.

Although several regional studies have examined either scoliosis prevalence or treatment satisfaction [6,7], none have comprehensively investigated both public awareness and social acceptance of screening and treatment options on a national scale. This study therefore aims to fill that gap by evaluating the level of scoliosis knowledge among the Saudi public, perceptions of school-based screening, and acceptance of various treatment approaches. In contrast to earlier work, the present study also explores the social and cultural determinants that influence these attitudes—providing a broader understanding of how awareness, acceptance, and health behavior intersect in the Saudi population.

## 2. Methodology

### 2.1. Study Design and Setting

A nationwide, cross-sectional survey was conducted between May and June 2025 across multiple public venues in Saudi Arabia, including shopping malls, schools, and King Khalid University Hospital (KKUH) in Riyadh. The study coincided with the 2025 Saudi National Scoliosis Awareness Campaign, allowing access to a wide public audience.

### 2.2. Participants and Sampling

Eligible participants were Saudi adults aged 18 years and older, including parents. Individuals younger than 18 years or healthcare professionals specializing in spine care were excluded. A convenience sampling approach was used, and all visitors at campaign sites were invited by trained research staff. Online participation was also encouraged to ensure regional diversity.

### 2.3. Data Collection Tools

Data were collected using a structured, self-administered questionnaire distributed in two formats: In-person: through tablets or QR codes displayed at campaign booths. Online: via electronic forms shared on social media platforms.

Participation was voluntary, anonymous, and uncompensated. Informed consent was obtained electronically before the survey began.

### 2.4. Questionnaire Description

The survey instrument titled the Scoliosis Awareness and Acceptance Questionnaire (SAAQ) was adapted from Alashgar et al. (2023) [8], which originally focused solely on scoliosis awareness. For the purposes of this study, the questionnaire was modified and expanded to additionally assess attitudes toward screening, acceptance of treatment options, and barriers, facilitators, and preferred information sources. The SAAQ now measures five domains: General awareness and knowledge, Symptom recognition and perceived causes, Attitudes toward screening, Acceptance of treatment options, Barriers, facilitators, and preferred information source.

The adaptation followed rigorous linguistic and cultural validation: forward translation into Arabic, back-translation into English, expert review by bilingual clinicians, and pilot testing for clarity and relevance.

### 2.5. Survey Administration and Data Quality

Responses were collected through Computer-Assisted Web Interviewing (CAWI) and on-site electronic forms. Duplicate submissions were prevented by unique device-ID tracking. Incomplete entries were automatically excluded before data export. All data were stored on secure, encrypted servers. Anonymity and confidentiality were maintained throughout.

### 2.6. Sample Size and Power

A post hoc power analysis indicated that a minimum of 1000 participants would provide 90% power to detect small to medium effect sizes (Cohen’s w = 0.1) at α = 0.05. The achieved sample of 2055 respondents exceeded this threshold, ensuring adequate statistical reliability.

### 2.7. Statistical Analysis

Data were analyzed using IBM SPSS Statistics, Version 22. Continuous variables were summarized as mean ± standard deviation, and categorical variables as frequencies and percentages. Between-group differences were assessed using the Chi-square test for categorical data. *p*-values < 0.05 were considered significant.

The study protocol was approved by the Institutional Review Board of the College of Medicine, King Saud University (IRB No. E-25-9753).

## 3. Results

A total of 2055 participants were included in the study, with a predominance of females (68.7%) compared to males (31.3%). The mean age was 32.58 years (SD ± 12.92), reflecting a relatively young adult cohort. Most respondents resided in the central region of Saudi Arabia (74.4%), with the remainder distributed across the northern, southern, eastern, and western regions. The sample was highly educated, with 61.3% holding a bachelor’s degree and 10.9% having completed graduate studies, while only 3.2% reported an education level of secondary or below. Over half of the participants (55.7%) reported not having children (Table 1).

General awareness of scoliosis was reported by 70.9% of respondents; however, only 18.1% rated their understanding as good, 43.9% considered it average, and 38.0% reported no knowledge. Awareness of scoliosis symptoms was reported by 38.5% of participants. Among these, 75.1% recognized spinal curvature, 62.3% cited back pain, and 43.6% noted uneven shoulders, while 66.9% selected “other”.

Regarding perceptions of affected populations, 51.3% believed scoliosis affects both sexes equally, and 33.3% identified females as more commonly affected. Reported causes included congenital (54.5%), idiopathic (35.3%), neuromuscular (22.6%), and spinal trauma (42.8%).

Treatment options most frequently recognized were surgical intervention (67.4%) and physical therapy (62.0%), followed by bracing (40.5%) and observation (22.9%). Only 14.8% reported a family history of scoliosis, and 9.3% had attended an awareness campaign. The most common initial sources of information were social media (34.6%), family and friends (19.9%), and healthcare providers (10.6%) (Table 2).

Most respondents (70.3%) believed that scoliosis awareness in their region was insufficient. The majority supported the organization of awareness campaigns in public spaces and educational institutions (91.8%) and endorsed the implementation of school-based screening programs (88.2%). The mean preferred screening age was 8.03 years (SD ± 5.56). Almost all respondents (92.1%) believed that early detection of scoliosis improves outcomes.

When presented with a hypothetical diagnosis of mild scoliosis (<25°), 30.5% considered observation an acceptable management plan, 35.1% disagreed, and 34.4% were uncertain. Bracing was more widely accepted, with 64.3% in favor, 8.4% opposed, and 27.3% undecided. Surgical intervention was accepted by 53.5% of respondents, while 11.6% rejected it and 34.9% were unsure. The most frequently reported concerns regarding bracing or surgery were pain (54.7%) and treatment effectiveness (50.4%), followed by appearance (30.9%), cost (22.2%), and social stigma (10.5%) (Table 3).

When asked about perceived barriers to scoliosis screening in Saudi Arabia, the most frequently reported concern was lack of awareness (68.2%), followed by difficulty accessing specialists (19.9%), cost (8.9%), and stigma (3.0%). The most commonly cited facilitators for encouraging screening participation included health education (33.1%), free screening services (25.1%), and school- or workplace-based screening (22.3%). Encouragement by healthcare providers was reported by 6.2% of respondents.

Healthcare professionals were the most preferred source for receiving information about scoliosis (79.2%), followed by social media (42.1%) and schools (32.9%). Respondents prioritized information on symptoms and early warning signs (61.7%), followed by methods for preventing condition progression (11.5%), when to seek medical attention (8.6%), and available treatment options (8.0%). In contrast, interest in other topics such as physical activity and sports, differences between scoliosis types, long-term treatment outcomes, local and global statistics, and information on bracing and surgery was much lower, each selected by only around 2% of participants (Table 4).

Among respondents under 25 years of age, 79.8% had heard about scoliosis compared to 64.0% in older age groups, a statistically significant difference (*p* < 0.001). In the younger group, 49.2% reported average knowledge and 22.0% reported good knowledge, while 45.9% of those aged 25 years and above were uncertain, also statistically significant (*p* < 0.001).

Knowledge of scoliosis symptoms, including uneven shoulders, spinal curvature, and back pain, was statistically significantly higher among participants under 25 years of age (*p* < 0.001). Approximately half of all respondents believed scoliosis affects both genders equally, with this perception most common among those aged 25–45 years (*p* < 0.001). Belief that scoliosis is curable was more frequent among participants under 25 years (71.0%) (*p* < 0.001), whereas 42.0% of participants over 45 years were uncertain.

The most frequently identified possible cause of scoliosis was congenital origin, followed by spinal trauma, idiopathic causes, and neuromuscular causes, with younger participants statistically significantly more likely to select these options (*p* < 0.001) (Table 5).

Regarding treatment options for scoliosis, surgery and physical therapy were the most frequently selected options, particularly among participants under 25 years of age, at 78.5% and 69.2%, respectively, both statistically significant (*p* < 0.001), while observation and bracing were less frequently chosen.

More than 65.0% of respondents reported no family history of scoliosis. Over 87.0% had never attended a scoliosis awareness event, with a statistically significant difference observed among participants over 45 years of age (*p* < 0.001). Over 30.0% of respondents first heard about scoliosis through social media. Among participants under 25 years of age, the most common initial source of information was social media, followed by schools (both statistically significant, *p* < 0.001), whereas those over 45 years most frequently cited family and friends (*p* < 0.001) (Table 5).

## 4. Discussion

This nationwide survey provides the first large-scale overview of public awareness, screening acceptance, and treatment attitudes toward scoliosis in Saudi Arabia. Although most participants had heard of scoliosis, true understanding of its causes, symptoms, and management was limited. Awareness was higher among younger respondents, reflecting growing digital exposure and participation in national health campaigns. The findings highlight a clear gap between recognition of scoliosis as a condition and actual knowledge of its detection and treatment an issue consistent with previous Saudi reports that documented limited public familiarity with musculoskeletal disorders [8].

The high level of support for school-based screening in this study underscores the public’s readiness for organized preventive programs. Nearly nine in ten respondents favored such screening and recognized the benefits of early detection. These results mirror international experiences where structured school screening has improved early referral and reduced surgical rates [9,10,11]. Public surveys report strong parental and community support, motivated by recognition of visible deformities and the benefits of early detection [12]. The preference for early screening among younger participants may indicate that awareness campaigns and social-media exposure are effectively shaping attitudes toward prevention. Still, the lack of standardized national screening programs in Saudi Arabia remains a missed opportunity for early intervention.

Public attitudes toward treatment showed a clear inclination toward active management. Bracing was the most accepted approach, followed by surgery, while observation alone was less favored. This pattern reflects both trust in medical intervention and cultural emphasis on visible action toward illness. However, reported concerns about pain, appearance, and uncertainty about brace effectiveness are consistent with psychosocial barriers described internationally [13,14,15]. The moderate acceptance of surgery also aligns with evidence that invasive procedures evoke anxiety and stigma in conservative societies [16]. Together, these findings suggest that patient-centered counseling, family involvement, and culturally sensitive education are essential to improve adherence and treatment satisfaction.

The role of digital media in shaping awareness was striking. Over one-third of participants first learned about scoliosis through social platforms, yet nearly four-fifths preferred to receive future information from healthcare professionals. This contrast indicates that while social media effectively spreads initial awareness, the public still trusts medical sources for credible education. Prior analyses of online scoliosis content have shown poor accuracy and overemphasis on self-image rather than health education [17,18]. Integrating healthcare-verified content into popular platforms could bridge this gap and combat misinformation. Awareness campaigns that combine school-based outreach with targeted digital strategies are likely to achieve the greatest impact [19].

Sociodemographic patterns also carry important implications. Females and younger respondents demonstrated higher awareness and acceptance of screening and treatment. Similar findings have been reported in regional and global studies [6,7,20]. In Saudi Arabia, this may stem from increased participation of young women in health education events, the influence of social media, and greater exposure to body-image discussions online. The generational gap in awareness suggests that campaigns must diversify outreach, extending beyond digital channels to reach older adults through community events and traditional media. Stigma, though reported by only a small minority, remains a relevant cultural barrier. Addressing it through normalization, positive role models, and community-based education can help improve both participation and treatment adherence.

From a public-health standpoint, the results indicate strong societal readiness for formal scoliosis screening and education policies. Participants overwhelmingly identified lack of awareness and access as key barriers, consistent with global evidence emphasizing education and affordability as central determinants of engagement [21,22,23,24]. Facilitators such as free screening, health education, and school involvement reflect practical, low-cost interventions that could be implemented within existing healthcare frameworks. Emerging digital tools such as tele-screening applications and mobile awareness platforms may also enhance accessibility and participation [25].

Based on these findings, several actions are recommended. First, scoliosis screening should be incorporated into the national school-health program, supported by standardized nurse and teacher training. Second, awareness campaigns should coincide with global events such as Scoliosis Awareness Month, coordinated between the Ministries of Health and Education [19,26]. Third, government and private stakeholders should subsidize brace treatment and screening services to ensure equitable access. Finally, scoliosis education should be embedded into health curricula to foster early literacy and normalize spinal-health discussions among youth [27,28].

Health literacy remains a critical factor influencing early recognition and treatment adherence. Studies show that while adolescents may recognize scoliosis, few understand its management or progression [27,28]. Bridging this gap through curriculum-based education, parental workshops, and interactive outreach will be vital to sustaining awareness gains. Regional variability in prevalence, with local estimates as high as 19%, further reinforces the need for a unified national framework for screening and public education [29].

Overall, this study provides a comprehensive, data-driven baseline for understanding scoliosis awareness and social acceptance in Saudi Arabia. The findings reveal a population that is motivated yet insufficiently informed, supportive of early detection but constrained by limited access and persistent misconceptions. Implementing culturally attuned, evidence-based policies could translate this willingness into measurable improvements in early diagnosis, brace compliance, and long-term outcomes.

### Public-Health and Policy Implications

The exceptionally high support for school-based screening (88%) demonstrates public readiness for structured national initiatives. The preferred screening age of eight years suggests trust in early pediatric assessment. Yet, the lack of awareness (68%) as the most cited barrier highlights that any screening policy must be preceded by comprehensive education campaigns.

This aligns with evidence from other countries where mandatory school screening, coupled with parental education, improved early detection and reduced surgical burden. Implementation in Saudi Arabia should therefore integrate teacher training, school nurse certification, and digital pre-screening tools to maximize feasibility and cost-effectiveness.

The findings carry clear implications for national policy:

- Integrate scoliosis screening into existing school-health programs.
- Train primary-care and school nurses to perform preliminary screening using Adam’s test and scoliometer.
- Launch bilingual educational campaigns emphasizing early recognition and treatment adherence.
- Subsidize bracing and screening costs to improve accessibility for low-income families.
- Collaborate with digital-media outlets to counter misinformation and promote professional health messaging.

## 5. Conclusions

This study highlights critical gaps in scoliosis knowledge among the Saudi public, despite strong support for school-based screening and national awareness campaigns. Bracing and surgery were more widely accepted than observation, though concerns about pain, effectiveness, and stigma remain important barriers. Social media is an influential source of initial information, yet healthcare professionals are the most trusted providers of future education. By addressing awareness gaps, reducing barriers to access, and integrating culturally sensitive education into schools and communities, policymakers and healthcare providers can strengthen early detection and improve scoliosis care in Saudi Arabia. Future research should assess the impact of school-based and digital interventions in enhancing scoliosis literacy and early recognition.

## 6. Limitations

This study has several limitations. First, its cross-sectional design precludes causal inferences between demographic factors, awareness, and treatment acceptability. Second, convenience sampling through campaign events and online platforms may introduce selection bias, as participants may have had greater health interest or access to information than the general population. In addition, the predominance of female respondents may have influenced findings related to awareness and attitudes, limiting the generalizability across genders. Third, reliance on self-reported data carries the risk of recall and social desirability bias. Fourth, although the questionnaire was culturally adapted and piloted, unmeasured variables such as prior exposure to healthcare or regional disparities may have influenced responses. Fifth, although participants expressed high acceptability for school-based scoliosis screening, their responses may not have been based on detailed knowledge of the specific screening methods used locally; thus, these results primarily reflect general attitudes and willingness to participate in preventive programs. Therefore, the findings may not be fully generalizable to the entire Saudi population. Despite these limitations, the large sample size and nationwide reach provide a valuable baseline for understanding public awareness and social acceptability of scoliosis screening and treatment in Saudi Arabia.

## Figures and Tables

**Table 1:** Demographic characteristics of study participants (N = 2055).

		Number	%
Gender	Male	643	31.3
	Female	1412	68.7
Age (Mean, SD)		32.58	12.92
Region of residence	Middle	1529	74.4
	North	92	4.5
	South	140	6.8
	West	144	7.0
	East	150	7.3
Education level	Elementary	10	0.5
	Intermediate	55	2.7
	Secondary	506	24.6
	Bachelor’s	1259	61.3
	Graduate	225	10.9
Do you have children?	Yes	910	44.3
	No	1145	55.7

**Table 2:** Knowledge, prior exposure, and experiences related to scoliosis.

		Number	%
Have you ever heard about scoliosis before?	Yes	1457	70.90
	No	598	29.10
How would you rate your knowledge about scoliosis?	Good	372	18.10
	Average	903	43.94
	I don’t know	780	37.96
Do you know the symptoms of scoliosis?	Yes	792	38.54
	No	1263	61.46
common symptoms of scoliosis	uneven shoulders	896	43.60
	curved spine	1543	75.09
	back pain	1280	62.29
	other	1375	66.91
Who do you think scoliosis affects more?	Females	685	33.33
	Males	315	15.33
	Both	1055	51.34
Do you think there is a cure for scoliosis?	Yes	1348	65.60
	No	85	4.14
	I don’t know	622	30.27
possible causes of scoliosis	idiopathic	726	35.33
	neuromuscular	464	22.58
	congenital	1120	54.50
	spinal trauma	879	42.77
	other	1169	56.89
treatment options for scoliosis	Observation	470	22.87
	Bracing	833	40.54
	Surgery	1385	67.40
	Physical therapy	1274	62.00
	Other	226	11.00
Has anyone in your family been diagnosed with scoliosis?	Yes	304	14.79
	No	1506	73.28
	I don’t know	245	11.92
Have you ever attended any campaign or activity regarding scoliosis awareness?	Yes	191	9.29
	No	1864	90.71
How did you first learn about scoliosis?	Social media	711	34.60
	school	257	12.51
	healthcare provider	218	10.61
	family/friends	410	19.95
	never heard	459	22.34

**Table 3:** Awareness, attitudes, acceptability, and treatment preferences for scoliosis.

		Number	%
Do you think there is enough awareness about scoliosis in your region?	Yes	157	7.64
	No	1445	70.32
	I don’t know	453	22.04
Do you support organizing exhibitions or campaigns in schools, universities, and public places to raise awareness about scoliosis?	Yes	1886	91.78
	No	169	8.22
Do you support the idea of school-based scoliosis screening programs?	Yes	1812	88.18
	No	89	4.33
	I don’t know	154	7.49
At what age do you think children should be screened for scoliosis? (Mean, SD)		8.03	5.56
Do you believe early detection of scoliosis can improve outcomes?	Yes	1892	92.07
	No	40	1.95
	I don’t know	123	5.99
If you, a family member, or someone close to you were diagnosed with mild scoliosis (<25°), would you consider observation alone an appropriate management plan?	Yes	626	30.46
	No	722	35.13
	Not sure	707	34.40
If a back brace was recommended as part of the treatment plan, would you find this option acceptable?	Yes	1321	64.28
	No	173	8.42
	Not sure	561	27.30
If surgery was recommended to treat scoliosis, would you accept this option for yourself or someone close to you?	Yes	1100	53.53
	No	238	11.58
	Not sure	717	34.89
What concerns would you have regarding bracing or surgery?	appearance	636	30.95
	pain	1123	54.65
	effectiveness	1036	50.41
	cost	457	22.24
	social stigma	215	10.46
	other	361	17.57

**Table 4:** Perceived barriers, facilitators, and information needs regarding scoliosis screening.

		Number	%
What do you think are the main barriers to scoliosis screening in Saudi Arabia?	Lack of awareness	1402	68.22
	cost	183	8.91
	stigma	62	3.02
	Difficulty accessing specialists	408	19.85
What factors would encourage you to participate in or support a screening program?	Encouragement from doctors or healthcare providers	111	6.15
	Health education	597	33.06
	Free screening	453	25.08
	School- or workplace-based screening	403	22.31
	Access to reliable information from official sources	96	5.32
	awareness campaigns	146	8.08
Where would you prefer to receive information about scoliosis?	Healthcare providers	1628	79.22
	schools	675	32.85
	social media	866	42.14
	TV/radio	266	12.94
	printed materials	237	11.53
What type of information would you like to receive?	Symptoms and early warning signs	1268	61.70
	Methods for preventing condition progression	237	11.53
	When to seek medical attention	177	8.61
	Available treatment options	164	7.98
	Physical activity and sports for people with scoliosis	47	2.29
	Differences between scoliosis types	46	2.24
	Long-term treatment outcomes	46	2.24
	Local and global statistics on scoliosis	41	2.00
	Information on bracing and surgery	29	1.41

**Table 5:** Scoliosis knowledge, prior exposure, and information sources by age group.

		<25 y (N = 884)		25–45 y (N = 748)		>45 y (N = 414)		*p* Value
		Number	%	Number	%	Number	%	
Have you ever heard about scoliosis before?	Yes	706	79.86	481	64.30	265	64.01	<0.001 *
	No	178	20.14	267	35.70	149	35.99	
How would you rate your knowledge about scoliosis?	Good	195	22.06	117	15.64	56	13.53	<0.001 *
	Average	435	49.21	298	39.84	168	40.58	
	I don’t know	254	28.73	333	44.52	190	45.89	
Do you know the symptoms of scoliosis?	Yes	433	48.98	250	33.42	106	25.60	<0.001 *
	No	451	51.02	498	66.58	308	74.40	
common symptoms of scoliosis	uneven shoulders	504	57.01	279	37.30	110	26.57	<0.001 *
	curved spine	750	84.84	505	67.51	282	68.12	<0.001 *
	back pain	638	72.17	438	58.56	199	48.07	<0.001 *
	other	659	74.55	478	63.90	231	55.80	<0.001 *
Who do you think scoliosis affects more?	Females	330	37.33	223	29.81	128	30.92	<0.001 *
	Males	157	17.76	99	13.24	58	14.01	
	Both	397	44.91	426	56.95	228	55.07	
Do you think there is a cure for scoliosis?	Yes	629	71.15	482	64.44	230	55.56	<0.001 *
	No	51	5.77	26	3.48	8	1.93	
	I don’t know	204	23.08	240	32.09	176	42.51	
possible causes of scoliosis	idiopathic	383	43.33	233	31.15	109	26.33	<0.001 *
	neuromuscular	238	26.92	150	20.05	75	18.12	
	congenital	581	65.72	370	49.47	165	39.86	<0.001 *
	spinal trauma	433	48.98	290	38.77	151	36.47	<0.001 *
	other	609	68.89	381	50.94	172	41.55	<0.001 *
treatment options for scoliosis	Observation	261	29.52	148	19.79	58	14.01	<0.001 *
	Bracing	458	51.81	262	35.03	111	26.81	<0.001 *
	Surgery	694	78.51	467	62.43	219	52.90	
	Physical therapy	612	69.23	435	58.16	221	53.38	<0.001 *
	Other	64	7.24	101	13.50	60	14.49	<0.001 *
Has anyone in your family been diagnosed with scoliosis?	Yes	109	12.33	117	15.64	76	18.36	<0.001 *
	No	698	78.96	531	70.99	270	65.22	
	I don’t know	77	8.71	100	13.37	68	16.43	
Have you ever attended any campaign or activity regarding scoliosis awareness?	Yes	112	12.67	51	6.82	26	6.28	<0.001 *
	No	772	87.33	697	93.18	388	93.72	
How did you first learn about scoliosis?	Social media	329	37.22	255	34.09	126	30.43	<0.001 *
	school	195	22.06	46	6.15	14	3.38	
	healthcare provider	98	11.09	87	11.63	31	7.49	
	family/friends	140	15.84	161	21.52	107	25.85	
	never heard	122	13.80	199	26.60	136	32.85	

* Significant *p* value.

## Data Availability

The datasets generated and analyzed during the current study are only available from the corresponding author on reasonable request in SPSS format due to ethical restrictions and the protection of participant confidentiality.

## Abbreviations

Scoliosis Awareness and Acceptance Questionnaire (SAAQ).
